# Design and Analysis of a 2-DOF Electromagnetic Actuator with an Improved Halbach Array for the Magnetic Suspension Platform

**DOI:** 10.3390/s22030790

**Published:** 2022-01-20

**Authors:** Fei Yang, Yong Zhao, Huaiyu Li, Xingke Mu, Wenqiao Zhang, Honghao Yue, Rongqiang Liu

**Affiliations:** 1 School of Mechatronics Engineering, Harbin Institute of Technology, Harbin 150080, China; yangf@hit.edu.cn (F.Y.); 20S008187@stu.hit.edu.cn (H.L.); yuehonghao@hit.edu.cn (H.Y.); liurq@hit.edu.cn (R.L.); 2 China Academy of Launch Vehicle Technology Research and Development Center, Beijing 100076, China; muxk@calt.casc; 3 Innovation Academy for Microsatellites of CAS, Shanghai 201210, China; zhangwq@microsate.com

**Keywords:** magnetic suspension platform, electromagnetic actuator, accurate subdomain model, measurement model, electromagnetic characteristics

## Abstract

For large bearing capacity and low current consumption of the magnetic suspension platform, a 2-DOF electromagnetic actuator with a new structure of halbach array is proposed to improve driving force coefficients. The structure and the working principle are introduced. An accurate sub domain model of the new structure is established to accurately and rapidly calculate the magnetic field distribution for obtaining the parameters and performance of the electromagnetic actuators. The analytical model results are verified by the finite element method. The force/torque model of the magnetic suspension platform is established based on the proposed 2-DOF electromagnetic actuator. Three position-sensitive detectors and six accelerometers are applied to perceive in real time the posture and vibration acceleration of the platform, respectively. Their hardware information is introduced and measurement models are established based on the layout. Finally, the electromagnetic characteristics of the proposed actuator are investigated and compared with the conventional counterpart by finite element analysis. The results show that the average magnetic field, 0.432 T, horizontal and vertical force coefficient, 92.3 N/A and 30.95 N/A, and torque in x and z direction, 3.61 N·m and 8.49 N·m, of the proposed actuator are larger than those of the conventional one.

## 1. Introduction

The microgravity environment of space stations has opened up a new frontier for experimental research in materials science, basic physics, life science, and biotechnology [1]. However, the conditions of acceleration-sensitive experiments are usually not satisfied because of the low-frequency and high-frequency micro-vibrations produced by gravity gradients, orbital maneuvers, attitude control, equipment operation, and astronaut activities in the space laboratory [2,3,4,5].

For decades, various researchers have made efforts to mitigate the effects of micro-vibrations [6,7,8]. Vibration isolation technology can be divided into passive isolation and active isolation according to the theory of vibration control. Passive isolation has a limited effort on low-frequency micro-vibration while the effect of active isolation is both significant to low-frequency and high-frequency micro-vibrations [9,10]. According to the driving force principle, the actuators used in active isolation are mainly electromagnetic, piezoelectric, phase-change, and magnetostrictive. The electromagnetic actuator has the advantages of large stroke, high resolution, high energy density, and so on. Without physical contact, the vibration of the base cannot be directly transmitted to the object, which is an effective approach to the isolation of micro-vibration in space [11,12,13]. It restricts the performance improvement for the vibration isolation platforms with multiple single DOF electromagnetic actuators due to the large size and mass, complex structure and control system, difficulty to miniaturize and compact [14,15]. While the 2-DOF electromagnetic actuators have obvious merits in the system cost and weight, layout optimization, and so on [16,17,18].

In Reference [19], suppression of transient accelerations by levitation driven by three 2-DOF electromagnetic actuators can provide a microgravity environment with an acceleration of 10^−6^ g. The stroke of each actuator is over ±10 mm in the horizontal and vertical directions. References [2,20] proposed a microgravity vibration isolation mount consisting of eight electromagnetic actuators with three 2-DOF displacement sensors and six accelerometers. It has a wide control bandwidth of 0.01 Hz~100 Hz. References [21,22] proposed a vibration isolation system consisting of three integrated vibration isolation units. Each isolation unit consisting of a 2-DOF electromagnetic actuator, two uniaxial accelerometers and an implicit two-axis displacement sensor, can achieve μg-class active isolation. It achieves characteristics of modularity and versatility through local control.

The electromagnetic actuator is the key component of the magnetic suspension vibration isolation system [23,24]. Reference [25] proposed a 2-DOF electromagnetic actuator that integrates horizontal and vertical driving. The movement range is ±5 mm in the horizontal direction while it is only 200 μm in the vertical direction. The size of the PM is 25.4 mm × 25.4 mm × 12.7 mm and the size of each winding is 40 mm × 40 mm × 17.5 mm. The volume of the permanent magnet and driving current of the winding is small, so the actuator has low energy consumption and heat loss, suitable for the miniaturized platform. Reference [26] put forward a 2-DOF electromagnetic actuator consisting of a permanent magnet and two windings as well. The rectangular and the cylindrical windings located below the axially magnetized cylindrical permanent magnet generate the horizontal and vertical driving forces respectively. The oversize is 60 mm × 60 mm × 40 mm and the movement range is 200 μm × 200 μm × 200 μm. In reference [25,26], only one permanent magnet is used to reduce volume, causing more magnetic leakage and lower utilization of magnetic field. Reference [17] proposed a 2-DOF electromagnetic actuator whose radial movement range is ±1 mm, axial movement range is ±5 mm, and oversize is 125 mm × 125 mm × 125 mm. Due to a new structure of hollow coil winding, the actuator achieves a compact arrangement of intersecting in space. Therefore, the structure of the actuator is compact and the mass is smaller. In reference [27], the mover floats in the vertical direction through the electromagnetic force produced by the e-type electromagnet. The actuation winding wound on the floater can drive the floater in the horizontal direction under the electromagnetic field, while the stroke in the vertical direction is small. Reference [28] proposed a 2-DOF electromagnetic actuator whose vertical and horizontal driving forces are produced through two sets of circuit board conductors perpendicular to each other in the same permanent magnetic field. The effective length of the conducting wires in the magnetic field and electromagnetic force is low. In reference [18], the 2-DOF electromagnetic actuator realizes the integration of horizontal and vertical driving forces and large strokes. However, it has some disadvantages such as large volume and mass, low driving force coefficient, and low deflection torque density.

In the control process of vibration isolation driven by an electromagnetic actuator, it is necessary to establish a position measurement model according to the layout characteristics of sensors, to acquire the real-time relative motion information between the base and the floating platform. Reference [17] proposed a measurement configuration using six displacement sensors, which are arranged between the driving units and measure the pose of the laser bracket in a noncontact way to make the structure more compact. References [21,22] proposed a patent-pending implicit position sensing technology, which installs a winding on the actuating winding to sense the relative motion of the actuator air gap. The system does not require additional lasers and displacement sensors, reducing the overall weight.

In order to realize active control of vibration isolation platforms, it is necessary to establish the linear acceleration and angular acceleration model of the platform according to the layout characteristics of the accelerometers. Reference [29] proposed an acceleration configuration using six accelerometers which are located at the center of the cube. The sensitive axes of accelerometers are diagonally oriented. The angular acceleration of the rigid body can be directly calculated from the measured values of accelerometers. Reference [30] proposed an acceleration measurement configuration of 12 linear accelerometers. The linear acceleration, angular acceleration, and angular velocity of the rigid body can be directly calculated without complicated calculation and derivation. In reference [31], a configuration of a planar triangle is designed to detect the 6-DOF acceleration. Six accelerometers are fixed to three vertices of an equilateral triangle, three of whose sensitive directions are vertical, and three of whose sensitive directions are tangent to the circumscribed circle of the equilateral triangle. This configuration has the advantages of compact structure, convenient installation, and small space. In reference [32], a three-axis integrated accelerometer, a two-axis integrated accelerometer, and a single-axis accelerometer mounted in the same plane are used to obtain acceleration of the platform and improve measurement precision.

The conventional 2-DOF electromagnetic actuators have some shortcomings, such as large size, magnetic leakage, low airgap magnetic field, and small driving force and torque coefficient. As to the pose and the acceleration measurement system, the layout and calculation methods are diversified for the magnetic levitation platform. In the paper, a novel 2-DOF electromagnetic actuator with an improved structure of halbach array is proposed to improve electromagnetic characteristics. It has the merits of high force coefficient and torque output, small volume, large force density, and low driving force fluctuation. This paper is organized as follows—the next section introduces the configuration and work principle of the proposed actuator and the platform. In Section 3, accurate magnet flux density model and electromagnetic force/torque models are established by subdomain method. In Section 4, position and accelerometer measurement models are established for the magnetic suspension platform with 2-DOF electromagnetic actuators. In Section 5, for highlighting the superiorities of the proposed halbach array structure, the magnetic field distribution, driving force, and torque are investigated and compared with conventional one through finite element method (FEM). To show the merits of the proposed electromagnetic actuators, the structure and performance parameters are compared with the typical one.

## 2. Structure and Working Principle

**Figure 1:**
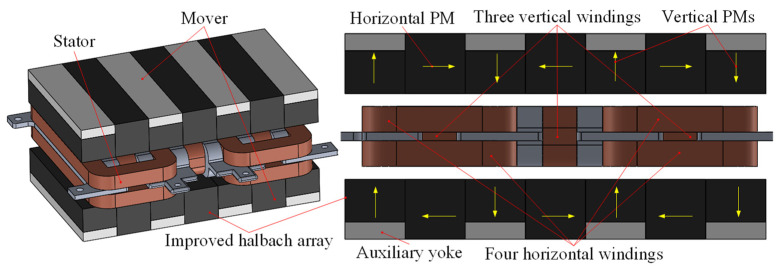
Configuration of the 2-DOF electromagnetic actuator.

**Figure 2:**
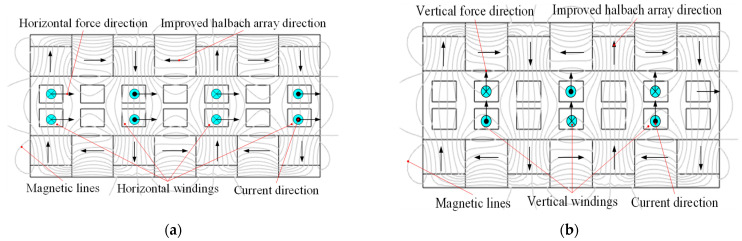
Working principle of the 2-DOF electromagnetic actuator: (**a**) Horizontal force; (**b**) Vertical force.

As shown in Figure 1, the two-degree-of-freedom electromagnetic actuator in this paper is mainly composed of the stator part and the mover part. The mover part mainly includes the upper and lower improved halbach permanent magnet (PM) arrays for providing airgap magnetic field in *x* and *y* directions. Different from the conventional structure of halbach array, the auxiliary yokes are added to the end of vertical PMs to reduce reluctance in the back region and improve airgap magnetic field. The stator part contains four horizontal windings and three vertical windings located in the airgap, corresponding to the horizontal and vertical PMs, respectively. Figure 2 shows the working principle of the 2-DOF electromagnetic actuator. Four horizontal coils carrying a direct current and exposed to the magnetic field generated by vertical PMs generate horizontal Ampere forces. Three vertical coils carrying a direct current and also exposed to the magnetic field produced by horizontal PMs generate vertical Ampere forces. Therefore, the proposed 2-DOF electromagnetic actuator integrates the vertical and horizontal driving forces.

**Figure 3:**
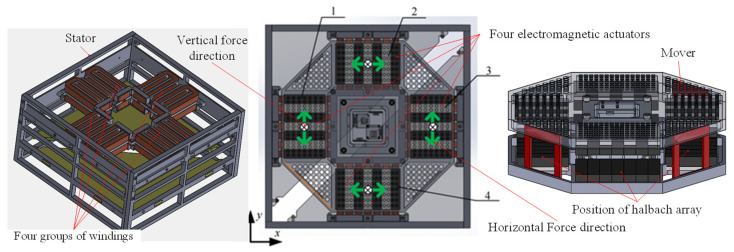
Structure and working principle of the magnetic suspension platform.

The structure of the magnetic suspension platform is shown in Figure 3. The magnetic levitation platform is mainly composed of a base and a floating platform. Four 2-DOF electromagnetic actuators are installed on the base in orthogonal distribution, and the mover part is connected to the platform floater. The mapping relationship between the platform motion and the driving force of 2-DOF actuators is shown in Table 1. The actuators 1 and 3 cooperate to drive the float to translate along the y direction and rotate around the y-axis. The actuators 2 and 4 cooperate to drive the float to translate along the *x* direction and rotate around the *x*-axis. And the actuators 1–4 work together to drive the float to translate along the z direction and rotate around the z-axis. Therefore, the 6-DOF floating platform can be supported and isolated from micro-vibration by four orthogonal 2-DOF electromagnetic actuators.

## 3. Electromagnetic Model

### 3.1. Subdomain Model

**Figure 4:**
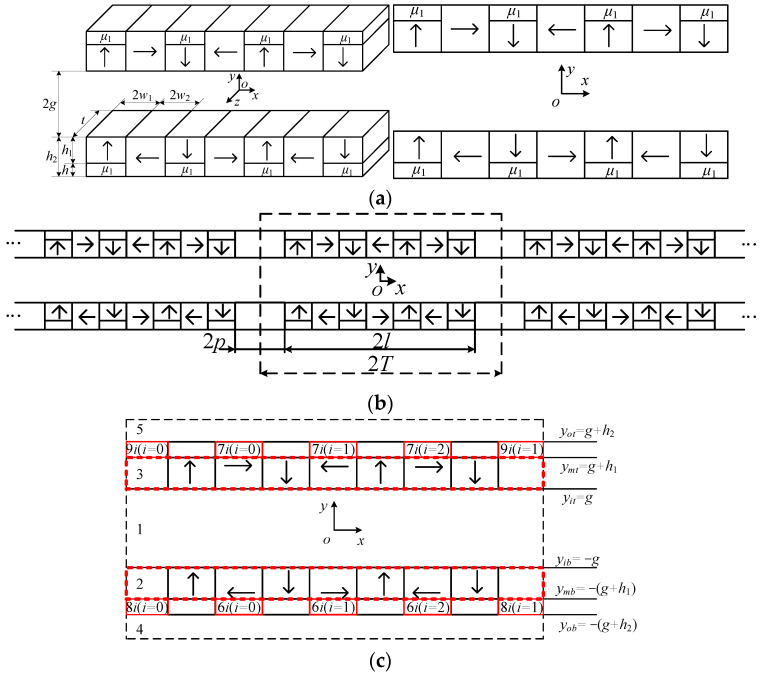
Equivalence process of the improved halbcah array structure: (**a**) Improved halbach array structure; (**b**) equivalent structure; (**c**) divided regions.

The equivalence process of improved halbcah array structure is shown in Figure 4. In order to obtain the magnetic field distribution at the end of the halbcah arrays, we expand the magnet arrays along the *x* direction as shown in Figure 4. The single-cycle structure of the improved halbach array is presented in the dotted line in Figure 4b. In order to solve the magnetic field distribution by subdomain method, the PM arrays with single cycle are divided into subdomains as shown in Figure 4c.

In the single-cycle subdomain model shown in Figure 4b, the relationship between $\vec{B}$ and $\vec{H}$ is satisfied as follows:

(1) $$\{\begin{array}{c} \vec{B}=\mu_{0}\vec{H}\;\;\;\mathrm{in}\;\mathrm{the}\;\mathrm{airgap}\\ \vec{B}=\mu_{0}\mu_{r}\vec{H}+\mu_{0}{\vec{M}}_{r}\;\mathrm{in}\;\mathrm{the}\;\mathrm{permanent}\;\mathrm{magnets} \end{array}$$

where ${\vec{M}}_{r}$ (A/m) is residual magnetization of a permanent magnet. $\mu_{0}$ (H/m) is vacuum permeability.

For the permanent magnet with linear demagnetization characteristic in the second quadrant, the amplitude of the residual magnetization vector $M_{r}$ is expressed as:

(2) $$M_{r}=\frac{B_{r}}{\mu_{0}}$$

The direction of ${\vec{M}}_{r}$ is determined by the magnetization direction and arrangement of the permanent magnet. In the cartesian coordinate system, the residual magnetization vector ${\vec{M}}_{r}$ can be expressed as:

(3) $${\vec{M}}_{r}=M_{x}\vec{i}+M_{y}\vec{j}$$

*M_x_*_3_ and *M_y_*_3_ are described by Fourier series.

(4) $$\left\{ \begin{array}{l} M_{x3}=\frac{a_{M0}}{2}+\sum_{n=1}^{\infty }a_{Mn}\cos\left(E_{n}x\right)\\ M_{y3}=\sum_{n=1}^{\infty }b_{Mn}\sin\left(E_{n}x\right) \end{array}\right.$$

where,

$$\begin{array}{l} a_{M0}=2M_{r}w_{2}/T,\;E_{n}=\frac{n\pi }{T}\\ a_{Mn}=\frac{2M_{r}}{n\pi }\left\{\sin\left[E_{n}\left(2w_{1}+3w_{2}\right)\right]-\sin\left[E_{n}\left(2w_{1}+w_{2}\right)\right]-\sin\left(E_{n}w_{2}\right)\right\}\\ b_{Mn}=\frac{2M_{r}}{n\pi }\left\{\cos\left[E_{n}l\right]-\cos\left[E_{n}\left(2w_{1}+3w_{2}\right)\right]-\cos\left[E_{n}\left(2w_{1}+w_{2}\right)\right]+\cos\left(E_{n}w_{2}\right)\right\} \end{array}$$

We can obtain magnetization of other regions as follows:

(5) $$\left\{ \begin{array}{l} M_{x2}=-M_{x3},\\ M_{y2}=M_{y3},\\ M_{x60}=-M_{x70}=-M_{r},\\ M_{y60}=M_{y70}=0,\\ M_{x61}=-M_{x71}=M_{r},\\ M_{y61}=M_{y71}=0,\\ M_{x62}=-M_{x72}=-M_{r},\\ M_{y62}=M_{y72}=0,\\ M_{x}=M_{y}=0 \end{array}\right.$$

The magnetic vectors for each region can be written as:

(6) $$\left\{ \begin{array}{l} A_{z1}=\sum_{n=0}^{\infty }\left[\left(A_{1n}e^{E_{n}y}+B_{1n}e^{-E_{n}y}\right)\cos\left(E_{n}x\right)+\left(C_{1n}e^{E_{n}y}+D_{1n}e^{-E_{n}y}\right)\sin\left(E_{n}x\right)\right]\\ A_{z2}=\sum_{n=0}^{\infty }\left[\left(A_{2n}e^{E_{n}y}+B_{2n}e^{-E_{n}y}\right)\cos\left(E_{n}x\right)+\left(C_{2n}e^{E_{n}y}+D_{2n}e^{-E_{n}y}\right)\sin\left(E_{n}x\right)\right]+\mu_{0}\sum_{n=1}^{\infty }b_{Mn}\frac{T}{n\pi }\cos\frac{n\pi x}{T}-\mu_{0}\frac{a_{M0}}{2}y\\ A_{z3}=\sum_{n=0}^{\infty }\left[\left(A_{3n}e^{E_{n}y}+B_{3n}e^{-E_{n}y}\right)\cos\left(E_{n}x\right)+\left(C_{3n}e^{E_{n}y}+D_{3n}e^{-E_{n}y}\right)\sin\left(E_{n}x\right)\right]+\mu_{0}\sum_{n=1}^{\infty }b_{Mn}\frac{T}{n\pi }\cos\frac{n\pi x}{T}+\mu_{0}\frac{a_{M0}}{2}y\\ A_{z4}=\sum_{n=0}^{\infty }\left[\left(A_{4n}e^{E_{n}y}+B_{4n}e^{-E_{n}y}\right)\cos\left(E_{n}x\right)+\left(C_{4n}e^{E_{n}y}+D_{4n}e^{-E_{n}y}\right)\sin\left(E_{n}x\right)\right]\\ A_{z5}=\sum_{n=0}^{\infty }\left[\left(A_{5n}e^{E_{n}y}+B_{5n}e^{-E_{n}y}\right)\cos\left(E_{n}x\right)+\left(C_{5n}e^{E_{n}y}+D_{5n}e^{-E_{n}y}\right)\sin\left(E_{n}x\right)\right]\\ A_{z6i}=\sum_{m=0}^{\infty }\left(A_{6im}e^{F_{m}y}+B_{6im}e^{-F_{m}y}\right)\cos\left[F_{m}\left(x-x_{i}+w_{2}\right)\right]+\mu_{0}M_{x6i}y\\ A_{z7i}=\sum_{m=0}^{\infty }\left(A_{7im}e^{F_{m}y}+B_{7im}e^{-F_{m}y}\right)\cos\left[F_{m}\left(x-x_{i}+w_{2}\right)\right]+\mu_{0}M_{x7i}y\\ A_{z8j}=\sum_{k=0}^{\infty }\left(A_{8jk}e^{H_{k}y}+B_{8jk}e^{-H_{k}y}\right)\cos\left[H_{k}\left(x-x_{j}+p\right)\right]\\ A_{z9j}=\sum_{k=0}^{\infty }\left(A_{9jk}e^{H_{k}y}+B_{9jk}e^{-H_{k}y}\right)\cos\left[H_{k}\left(x-x_{j}+p\right)\right] \end{array}\right.$$

The normal component of the magnetic induction and the tangential component of the magnetic field strength are continuous at the interface of different media. According to the boundary conditions among subdomains, *n* × (*B*_1_ − *B*_2_) = 0, *n* × (*H*_1_ − *H*_2_) = *J* × *s* and $\vec{B}=\nabla \times \vec{A}$, the flux density in each region can be solved.

**Figure 5:**
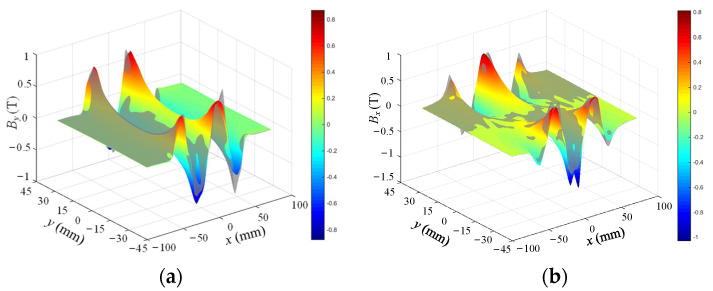
Comparison of analytical and simulation results of magnetic flux density: (**a**) y component; (**b**) *x* component.

The finite element method is used to verify the analytical model as shown in Figure 5. The results show that the analytical model is consistent with the simulation results. So, the analytical model correctly describes the magnetic field of the proposed halbach array.

### 3.2. Electromagnetic Force Model

**Figure 6:**
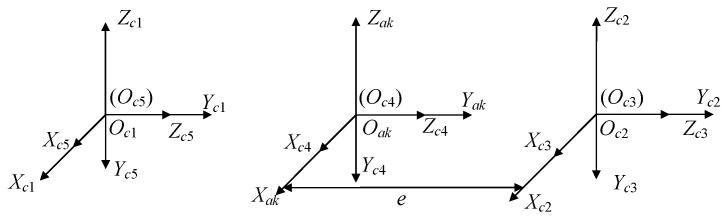
The coil array fixed coordinate system and the single coil fixed coordinate system.

The electromagnetic force is calculated in the fixed coordinate system of a single coil. The relationship between the coil array fixed coordinate system and the single coil fixed coordinate system is shown in Figure 6, e=(Hv+Wh)/2+Ws+Th.

The attitude transformation matrix from the coil array fixed coordinate system to every single coil is expressed as:

(7) {OakXakYakZak→OcjXcjYcjZcj:Rakcj =I3×3   j=1, 2OakXakYakZak→OcjXcjYcjZcj:Rakcj=[10000−1010] j=3, 4, 5

**Figure 7:**
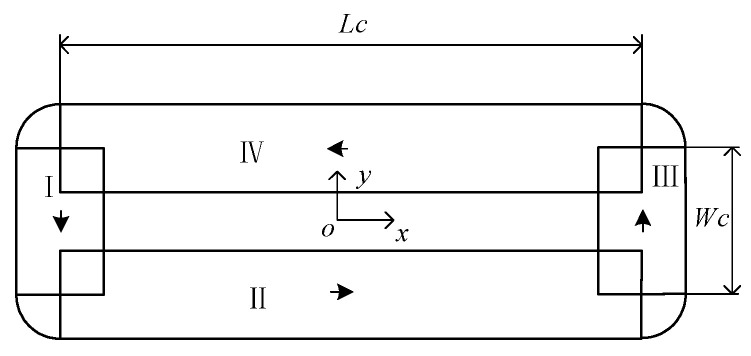
Simplified coil model.

The magnetic field is small at the corner of the windings. Therefore, it is simplified as a 4-segment cuboid current-carrying conductor by ignoring the factors such as winding fillet and uneven winding of the close-wound coil, as Figure 7 shows.

We take the j coil of the k coil array as the analysis object to conduct the force-current model. When the current is directed in a right-handed spiral with respect to the z axis of the single coil, the Lorentz force, ${\vec{F}}_{kj}$, can be expressed as:

(8) $${\vec{F}}_{kj}{}^{\left(cj\right)}=\sum_{n=Ⅰ,Ш}\underset{}{∭}\left(J_{kj}{\vec{e}}_{y}\right)\times {\vec{B}}_{n}{}^{\left(cj\right)}dV+\sum_{n=П,ІV}\underset{}{∭}J_{kj}{\vec{e}}_{x}\times {\vec{B}}^{\left(cj\right)}dV$$

where ${\vec{B}}^{\left(cj\right)}$(T) is magnetic flux density in the coordinate system, *cj*. $J_{kj}$(A/m^2^) is the volume current density of the *j* coil of the *k* array of winding.

A position vector ${\vec{r}}_{cj}$ in the coordinate system, *cj*, satisfies the following relationship:

(9) $${\vec{r}}_{cj}={\vec{r}}_{cjak}+{\vec{r}}_{akb}+{\vec{r}}_{bp}+{\vec{r}}_{pmi}+{\vec{r}}_{mi}$$

where ${\vec{r}}_{cjak}$ is the position vector from the origin of the single coil fixed coordinate system to the origin of the coil array fixed coordinate system, ${\vec{r}}_{akb}$ is the position vector from the origin of the single coil array fixed coordinate system to the origin of the base fixed coordinate system, ${\vec{r}}_{bp}$ is the position vector from the origin of the base fixed coordinate system to the origin of the floating platform fixed coordinate system, ${\vec{r}}_{pmi}$ is the position vector from the origin of the fixed coordinate system of the floating platform to the origin of the magnet array fixed coordinate system, ${\vec{r}}_{mi}$ is a position vector corresponding to ${\vec{r}}_{cj}$ in *mi* coordinate system.

${\vec{r}}_{mi}{}^{\left(mi\right)}$ can be further reduced to:

(10) r→mi(mi)=Rcjmir→cj(cj)+Rakmir→akcj(ak)+Rbmir→bak(b)−Rbmir→bp(b)−Rpmir→pmi(p)=Rcjmir→cj(cj)+p→akcj(mi)

where ${\vec{p}}_{akcj}{}^{\left(mi\right)}$ is the offset vector of the coordinate system, *cj*, relative to the coordinate system, *mi*.

In the coordinate system, *cj*, the magnetic flux density, ${\vec{B}}^{\left(cj\right)}$ can be described as:

(11) B→n(cj)=RmicjB→n(mi)(Rcjmir→cj(cj)+p→akcj(mi))

where ${\vec{B}}^{\left(mi\right)}\left({\vec{r}}_{mi}{}^{\left(mi\right)}\right)$ is the magnetic field in the magnet array fixed coordinate system.

From Rcjmi=RpmiRbpRaibRcjai (i=1, 2, 3, 4; j=1, 2, 3, 4, 5), the Rcjm1, Rcjm2, Rcjm3 and Rcjm4 can be solved. From p→akcj(mi)=Rakmir→akcj(ak)+Rbmir→bak(b)−Rbmir→bp(b)−Rpmir→pmi(p) and ${\vec{r}}_{bp}{}^{\left(b\right)}={\left[\begin{array}{ccc} X & Y & Z \end{array}\right]}^{T}$, the ${\vec{p}}_{a1cj}{}^{\left(m1\right)}$, ${\vec{p}}_{a2cj}{}^{\left(m2\right)}$, ${\vec{p}}_{a2cj}{}^{\left(m3\right)}$ and ${\vec{p}}_{a2cj}{}^{\left(m4\right)}$ can be obtained.

Submitting Equation (11) into Equation (8), ${\vec{F}}_{kj}$ could be simplified as follows:

(12) $${\vec{F}}_{kj}{}^{\left(cj\right)}={\vec{K}}_{kj}{}^{\left(cj\right)}J_{kj}$$

where,

K→kj(cj)=Rmicj{e→x×∑n=П,ІV∭B→n(mi)(Rcjmir→cj(cj)+p→akcj(mi))dV+e→y×∑n=Ⅰ,Ш∭B→n(mi)(Rcjmir→cj(cj)+p→akcj(mi))dV} 

The driving force on the platform float is expressed as:

(13) $${\vec{F}}^{\left(cj\right)}=-\sum_{k=1}^{4}\sum_{j=1}^{5}{\vec{F}}_{kj}{}^{\left(cj\right)}$$

Based on the simplified coil model shown in Figure 7, the electromagnetic torque is calculated in a single-coil fixed coordinate system.

Taking the *j* coil of the *k* winding array as the analysis object, the torque, ${\vec{M}}_{kj}{}^{\left(cj\right)}$, around the origin, $O_{p}$, of the fixed coordinate system of the platform float is expressed as:

(14) $${\vec{M}}_{kj}{}^{\left(cj\right)}=\sum_{n=І,Ш}\underset{}{∭}{\vec{r}}_{pkcj}{}^{\left(cj\right)}\times \left[\left(J_{kj}{\vec{e}}_{y}\right)\times {\vec{B}}_{n}{}^{\left(cj\right)}\right]dV+\sum_{n=П,ІV}\underset{}{∭}{\vec{r}}_{pkcj}{}^{\left(cj\right)}\times \left[J_{kj}{\vec{e}}_{x}\times {\vec{B}}_{n}{}^{\left(cj\right)}\right]dV$$

where ${\vec{r}}_{pkcj}$ is the position vector from the origin of the fixed coordinate system of the floating platform to a point of the winding.

${\vec{r}}_{pkcj}$ can be denoted as:

(15) r→pkcj(cj)=−Rbcjr→bp(b)+Rbcjr→bak(b)+Rakcjr→akcj(ak)+r→cj(cj)

Submitting (15) into (14), the torque, ${\vec{M}}_{kj}{}^{\left(cj\right)}$, is simplified to:

(16) $${\vec{M}}_{kj}{}^{\left(cj\right)}={\vec{N}}_{kj}{}^{\left(cj\right)}J_{kj}$$

where,

N→kj(cj)=(−Rbcjr→bp(b)+Rbcjr→bak(b)+Rakcjr→akcj(ak))×K→kj(cj)+Rmicj[∑n=І,Ш∭yB→n(mi)(Rcjmir→cj(cj)+p→akcj(mi))dV−∑n=П,ІV∭xB→n(mi)(Rcjmir→cj(cj)+p→akcj(mi))dV] +[∑n=П,ІV∭r→cj(mi)·B→n(mi)(Rcjmir→cj(cj)+p→akcj(mi))dV]e→x+[∑n=І,Шr→cj(mi)·B→n(mi)(Rcjmir→cj(cj)+p→akcj(mi))dV]e→y

According to Newton’s third law, the driving torque acting on the platform float is:

(17) $${\vec{M}}^{\left(cj\right)}=-\sum_{k=1}^{4}\sum_{j=1}^{5}{\vec{M}}_{kj}{}^{\left(cj\right)}$$

## 4. Measurement Model for the Platform

Micro-vibration has the feature of small amplitude, low frequency, weak signal and easily interfered by external environment and signal. The acquisition and processing of the information of micro-vibration magnitude to form effective information feedback is an important step in the active isolation and suppression of micro-vibration, which also puts forward higher requirements for hardware systems. In the active vibration control system, comparing the obtained acceleration signals of the floating and the fixed platforms is not only the most direct method to evaluate the vibration suppression effect, but also provides the acceleration signal feedback for the closed-loop control system.

**Figure 8:**
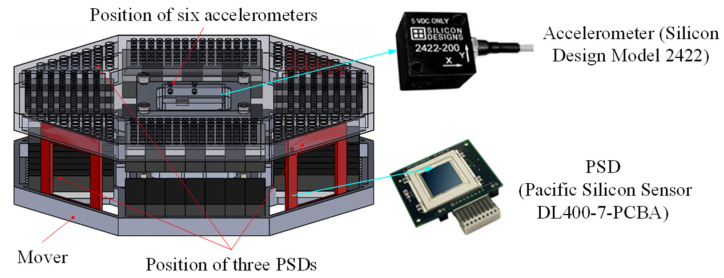
Accelerometers and PSDs on the mover.

As shown in Figure 8, the accelerometer in the paper is Model 2422 of Silicon Design with a range of ±2 g and a sensitivity of 2000 mV/g. The laser light sources and PSDs are applied to measure the relative position and attitude between the mover and the stator. The laser light sources are installed on the stator and the PSDs are fixed on the mover. The spot of the laser light sources is always within the range of the photosensitive surface of the PSDs. And the PSD could reflect the two-dimensional location information of the spot center on the photosensitive surface through outputting different analog voltages. The PSD in the paper is DL400-7-PCBA of Pacific Silicon Sensor with a range of ±20 mm and a resolution of about 1 μm.

### 4.1. Position Measurement Model

A six-dimensional position and attitude measurement model is established according to the installation position of sensors.

**Figure 9:**
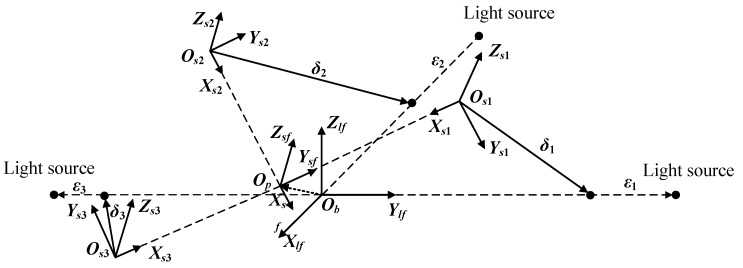
Coordinate system for six-dimensional position and orientation measurement model.

The PSD photosensitive plane coordinate system fixed with the tested platform, $O_{si}X_{si}Y_{si}Z_{si}\left(i=1,\;2,\;3\right)$, the laser reference coordinate system fixed with the base, $O_{b}X_{lf}Y_{lf}Z_{lf}$, and the photosensitive plane coordinate system fixed with the tested platform, $O_{p}X_{sf}Y_{sf}Z_{sf}$ are defined respectively in Figure 9. When the reference coordinate system of the laser and the reference coordinate system of the photosensitive surface coincide, the light spot is located at the origin of the coordinate system of each PSD.

The attitude transformation matrix from the fixed coordinate system of the platform float, $O_{p}X_{p}Y_{p}Z_{p}$, to the reference coordinate system of the photosensitive surface, $O_{p}X_{sf}Y_{sf}Z_{sf}$, can be described as:

(18) Rpsf=[22220−22220001]

The attitude transformation matrix from the fixed coordinate system of the base, $O_{b}X_{b}Y_{b}Z_{b}$, to the reference coordinate system of the laser, $O_{b}X_{lf}Y_{lf}Z_{lf}$, can be denoted as:

(19) Rblf=Rpsf

The attitude transformation matrix from the reference coordinate system of the photosensitive surface, $O_{p}X_{sf}Y_{sf}Z_{sf}$, to the coordinate system of each PSD photosensitive surface, $O_{si}X_{si}Y_{si}Z_{si}\left(i=1,\;2,\;3\right)$, can be described as:

(20) Rsfs1=[0−10100001],Rsfs2=I3×3,Rsfs3=[010−100001]

The coordinate arrays of each laser in the fixed coordinate system of the base are as follows:

(21) $${\vec{r}}_{bl1}{}^{\left(b\right)}={\left[\begin{array}{ccc} -a & a & 0 \end{array}\right]}^{T},{\vec{r}}_{bl2}{}^{\left(b\right)}={\left[\begin{array}{ccc} -a & -a & 0 \end{array}\right]}^{T},{\vec{r}}_{bl3}{}^{\left(b\right)}={\left[\begin{array}{ccc} a & -a & 0 \end{array}\right]}^{T}$$

The coordinate arrays of each photosensitive surface in the fixed coordinate system of the platform are as follows:

(22) $${\vec{r}}_{ps1}{}^{\left(p\right)}={\left[\begin{array}{ccc} -b & b & 0 \end{array}\right]}^{T},{\vec{r}}_{ps2}{}^{\left(p\right)}={\left[\begin{array}{ccc} -b & -b & 0 \end{array}\right]}^{T},{\vec{r}}_{ps3}{}^{\left(p\right)}={\left[\begin{array}{ccc} b & -b & 0 \end{array}\right]}^{T}$$

According to geometric relations and vector algorithms, we can obtain the equation as follow:

(23) $${\vec{r}}_{bp}+{\vec{r}}_{psi}+{\vec{\delta }}_{i}+{\vec{\varepsilon }}_{i}={\vec{r}}_{bli}$$

where ${\vec{r}}_{psi}$ is the position vector from $O_{p}$ to $O_{si}$ denoted as ${\vec{r}}_{ps1}{}^{\left(sf\right)}={\left[\begin{array}{ccc} 0 & \sqrt{2}b & 0 \end{array}\right]}^{T}$, ${\vec{r}}_{ps2}{}^{\left(sf\right)}={\left[\begin{array}{ccc} -\sqrt{2}b & 0 & 0 \end{array}\right]}^{T}$, ${\vec{r}}_{ps3}{}^{\left(sf\right)}={\left[\begin{array}{ccc} 0 & -\sqrt{2}b & 0 \end{array}\right]}^{T}$ in the reference coordinate system of the photosensitive surface, $O_{p}X_{sf}Y_{sf}Z_{sf}$. ${\vec{r}}_{bli}$ is the position vector from $O_{b}$ to $O_{li}$ denoted as ${\vec{r}}_{bl1}{}^{\left(lf\right)}={\left[\begin{array}{ccc} 0 & \sqrt{2}a & 0 \end{array}\right]}^{T}$, ${\vec{r}}_{bl2}{}^{\left(lf\right)}={\left[\begin{array}{ccc} -\sqrt{2}a & 0 & 0 \end{array}\right]}^{T}$, ${\vec{r}}_{bl3}{}^{\left(lf\right)}=[0{\begin{array}{cc} -\sqrt{2}a & 0 \end{array}]}^{T}$ in the reference coordinate system of the laser, $O_{b}X_{lf}Y_{lf}Z_{lf}$. ${\vec{\delta }}_{i}$ is the position vector from $O_{si}$ to the center of the light spot denoted as ${\vec{\delta }}_{i}{}^{\left(si\right)}=\left[\begin{array}{ccc} 0 & \delta_{yi} & \delta_{zi} \end{array}\right]$ in the corresponding coordinate system of the photosensitive surface, $O_{si}X_{si}Y_{si}Z_{si}$. ${\vec{\varepsilon }}_{i}$ is the position vector from light spot to light source denoted as ${\vec{\varepsilon }}_{1}{}^{\left(lf\right)}={\left[\begin{array}{ccc} 0 & \varepsilon_{1} & 0 \end{array}\right]}^{T}$, ${\vec{\varepsilon }}_{2}{}^{\left(lf\right)}={\left[\begin{array}{ccc} -\varepsilon_{2} & 0 & 0 \end{array}\right]}^{T}$, ${\vec{\varepsilon }}_{3}{}^{\left(lf\right)}=[0{\begin{array}{cc} -\varepsilon_{3} & 0 \end{array}]}^{T}$ in the reference coordinate system of the laser, $O_{b}X_{lf}Y_{lf}Z_{lf}$. ${\vec{r}}_{bp}$ is the position vector from $O_{b}$ to $O_{p}$ denoted as ${\vec{r}}_{bp}{}^{\left(lf\right)}={\left[\begin{array}{ccc} {\bar{p}}_{x} & {\bar{p}}_{y} & {\bar{p}}_{z} \end{array}\right]}^{T}$ in the reference coordinate system of the laser, $O_{b}X_{lf}Y_{lf}Z_{lf}$.

Equation (23) can be projected in the reference coordinate system of the laser to:

(24) r→bp(lf)+Rsflf(r→psi(sf)+Rsisfδ→i(si))+ε→i(lf)=r→bli(lf)

Cardan Angle is selected to describe the rotation of sf coordinate relative to *lf* coordinate. Rsflf can be denoted as:

(25) Rsflf=[cosβcosγ−cosβsinγsinβcosαsinγ+sinαsinβcosγcosαcosγ−sinαsinβsinγ−sinαcosβsinαsinγ−cosαsinβcosγsinαcosγ−cosαsinβsinγcosαcosβ]

where α is the angle of rotation around *x*_0_ axis. γ is the angle of rotation around *z*_2_ axis.

Submitting Equation (25) and relevant position vectors to Equation (24), β, α, ${\bar{p}}_{x}$, ${\bar{p}}_{y}$ and ${\bar{p}}_{z}$ can be solved as:

(26) $$\left\{ \begin{array}{l} {\bar{p}}_{x}=\sqrt{2}b\cos\beta sin\gamma -\delta_{y1}\cos\beta \cos\gamma -\delta_{z1}\sin\beta \\ {\bar{p}}_{y}=\sqrt{2}b\left(\cos\alpha \sin\gamma +\sin\alpha \sin\beta \cos\gamma \right)+\delta_{z2}\sin\alpha \cos\beta -\delta_{y2}\left(\cos\alpha \cos\gamma -\sin\alpha \sin\beta \sin\gamma \right)\\ {\bar{p}}_{z}=\left(\sqrt{2}bk_{2}-\delta_{y2}k_{1}-\delta_{z2}\right)\cos\alpha \cos\beta \\ \tan\alpha \cos\gamma -\sin\beta \sin\gamma =k_{1}\cos\beta \\ \left(\delta_{y1}+\delta_{y3}\right)\cos\gamma -2\sqrt{2}bsin\gamma +\left(\delta_{z1}-\delta_{z3}\right)\tan\beta =0\\ k_{2}\cos\gamma -k_{1}\sin\gamma +\tan\beta \cos\left(2\gamma \right)=0 \end{array}\right.$$

where,

$$k_{1}=\frac{\left(\sqrt{2}b+\delta_{y1}\right)\left(\delta_{z3}-\delta_{z2}\right)-\left(\sqrt{2}b-\delta_{y3}\right)\left(\delta_{z1}-\delta_{z2}\right)}{\left(\sqrt{2}b-\delta_{y2}\right)\left(\sqrt{2}b-\delta_{y3}\right)+\left(\sqrt{2}b+\delta_{y1}\right)\left(\sqrt{2}b+\delta_{y2}\right)},\;k_{2}=\frac{\delta_{z2}-\delta_{z1}+\left(\delta_{y2}-\sqrt{2}b\right)k_{1}}{\sqrt{2}b+\delta_{y1}}$$

When $\delta_{z1}\neq \delta_{z3}$:

(27) $$\left\{ \begin{array}{l} \tan\beta =k_{3}\cos\gamma -k_{4}\sin\gamma \\ k_{2}\cos\gamma -k_{1}\sin\gamma +\left(k_{3}\cos\gamma -k_{4}\sin\gamma \right)\cos\left(2\gamma \right)=0 \end{array}\right.$$

where,

$$k_{3}=\frac{\delta_{y1}+\delta_{y3}}{\delta_{z3}-\delta_{z1}},\;k_{4}=\frac{2\sqrt{2}b}{\delta_{z3}-\delta_{z1}}$$

When $\delta_{z1}=\delta_{z3}$:

(28) $$\left\{ \begin{array}{l} \gamma =\arctan\left(\frac{\delta_{y1}+\delta_{y3}}{2\sqrt{2}b}\right)\\ k_{2}\cos\gamma -k_{1}\sin\gamma +\tan\beta \cos\left(2\gamma \right)=0 \end{array}\right.$$

### 4.2. Accelerometer Measurement Model

**Figure 10:**
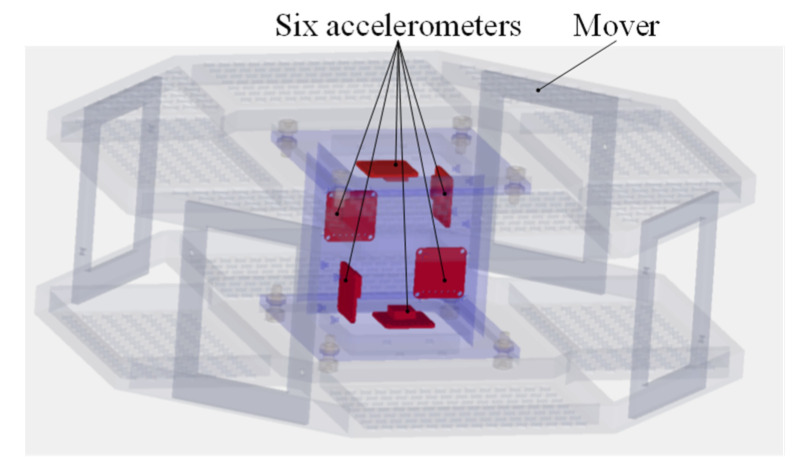
Detailed position of six accelerometers.

The detailed position of the six accelerometers is shown in Figure 10. The measurement result of the accelerometer is usually the acceleration of its stationary object relative to an inertial system. For simplification, it is assumed that the measurement results are from acceleration values relative to the inertial coordinate system of the spacecraft.

The output of an accelerometer installed on a floating platform at ${\vec{r}}_{i}$ with a sensing axis in the direction of ${\vec{\theta }}_{i}$. According to mounting position of six accelerometers, the sensitive axis directions of each accelerometer can be described as:

(29) $$A_{i}=\left[{\vec{a}}_{p}+\vec{\alpha }\times {\vec{r}}_{i}+\vec{\omega }\times \left(\vec{\omega }\times {\vec{r}}_{i}\right)\right]\cdot {\vec{\theta }}_{i}={\left[\begin{array}{c} \theta_{xi}\\ \theta_{yi}\\ \theta_{zi}\\ {\left({\vec{r}}_{i}\times {\vec{\theta }}_{i}\right)}_{x}\\ {\left({\vec{r}}_{i}\times {\vec{\theta }}_{i}\right)}_{y}\\ {\left({\vec{r}}_{i}\times {\vec{\theta }}_{i}\right)}_{z} \end{array}\right]}^{T}\left[\begin{array}{c} a_{px}\\ a_{py}\\ a_{pz}\\ \alpha_{x}\\ \alpha_{y}\\ \alpha_{z} \end{array}\right]+{\left[\begin{array}{c} -\theta_{yi}y_{i}-\theta_{zi}z_{i}\\ -\theta_{xi}x_{i}-\theta_{zi}z_{i}\\ -\theta_{xi}x_{i}-\theta_{yi}y_{i}\\ \theta_{yi}x_{i}+\theta_{xi}y_{i}\\ \theta_{zi}x_{i}+\theta_{xi}z_{i}\\ \theta_{zi}y_{i}+\theta_{yi}z_{i} \end{array}\right]}^{T}\left[\begin{array}{c} \omega_{x}{}^{2}\\ \omega_{y}{}^{2}\\ \omega_{z}{}^{2}\\ \omega_{x}\omega_{y}\\ \omega_{x}\omega_{z}\\ \omega_{y}\omega_{z} \end{array}\right]$$

The position coordinates of six accelerometer measurement point obtained are follows:

(30) $$\left\{ \begin{array}{l} {\vec{r}}_{1}={\left[\begin{array}{ccc} {\bar{x}}_{1} & {\bar{y}}_{1} & {\bar{z}}_{1} \end{array}\right]}^{T},\;{\vec{r}}_{2}={\left[\begin{array}{ccc} {\bar{x}}_{2} & {\bar{y}}_{2} & {\bar{z}}_{2} \end{array}\right]}^{T},\;{\vec{r}}_{3}={\left[\begin{array}{ccc} {\bar{x}}_{3} & {\bar{y}}_{3} & {\bar{z}}_{3} \end{array}\right]}^{T}\\ {\vec{r}}_{4}={\left[\begin{array}{ccc} {\bar{x}}_{4} & {\bar{y}}_{4} & {\bar{z}}_{4} \end{array}\right]}^{T},\;{\vec{r}}_{5}={\left[\begin{array}{ccc} {\bar{x}}_{5} & {\bar{y}}_{5} & {\bar{z}}_{5} \end{array}\right]}^{T},\;{\vec{r}}_{6}={\left[\begin{array}{ccc} {\bar{x}}_{6} & {\bar{y}}_{6} & {\bar{z}}_{6} \end{array}\right]}^{T} \end{array}\right.$$

Submitting Equation (30) into Equation (29), we can obtain:

(31) $$\left[\begin{array}{c} A_{1}\\ A_{2}\\ A_{3}\\ A_{4}\\ A_{5}\\ A_{6} \end{array}\right]=\left[\begin{array}{cccccc} -1 & 0 & 0 & 0 & -{\bar{z}}_{1} & {\bar{y}}_{1}\\ 1 & 0 & 0 & 0 & {\bar{z}}_{2} & -{\bar{y}}_{2}\\ 0 & -1 & 0 & {\bar{z}}_{3} & 0 & -{\bar{x}}_{3}\\ 0 & 1 & 0 & -{\bar{z}}_{4} & 0 & {\bar{x}}_{4}\\ 0 & 0 & -1 & -{\bar{y}}_{5} & {\bar{x}}_{5} & 0\\ 0 & 0 & 1 & {\bar{y}}_{6} & -{\bar{x}}_{6} & 0 \end{array}\right]\left[\begin{array}{c} a_{px}\\ a_{py}\\ a_{pz}\\ \alpha_{x}\\ \alpha_{y}\\ \alpha_{z} \end{array}\right]\;+\left[\begin{array}{cccccc} 0 & {\bar{x}}_{1} & {\bar{x}}_{1} & -{\bar{y}}_{1} & -{\bar{z}}_{1} & 0\\ 0 & -{\bar{x}}_{2} & -{\bar{x}}_{2} & {\bar{y}}_{2} & {\bar{z}}_{2} & 0\\ {\bar{y}}_{3} & 0 & {\bar{y}}_{3} & -{\bar{x}}_{3} & 0 & -{\bar{z}}_{3}\\ -{\bar{y}}_{4} & 0 & -{\bar{y}}_{4} & {\bar{x}}_{4} & 0 & {\bar{z}}_{4}\\ {\bar{z}}_{5} & {\bar{z}}_{5} & 0 & 0 & -{\bar{x}}_{5} & -{\bar{y}}_{5}\\ -{\bar{z}}_{6} & -{\bar{z}}_{6} & 0 & 0 & {\bar{x}}_{6} & {\bar{y}}_{6} \end{array}\right]\left[\begin{array}{c} \omega_{x}{}^{2}\\ \omega_{y}{}^{2}\\ \omega_{z}{}^{2}\\ \omega_{x}\omega_{y}\\ \omega_{x}\omega_{z}\\ \omega_{y}\omega_{z} \end{array}\right]$$

Because the platform float is actively controlled by 2-DOF electromagnetic actuators and its rate is small, the square term and cross-product term can be ignored. So, Equation (31) can be simplified as:

(32) $$\left[\begin{array}{c} A_{1}\\ A_{2}\\ A_{3}\\ A_{4}\\ A_{5}\\ A_{6} \end{array}\right]=\left[\begin{array}{cccccc} -1 & 0 & 0 & 0 & -{\bar{z}}_{1} & {\bar{y}}_{1}\\ 1 & 0 & 0 & 0 & {\bar{z}}_{2} & -{\bar{y}}_{2}\\ 0 & -1 & 0 & {\bar{z}}_{3} & 0 & -{\bar{x}}_{3}\\ 0 & 1 & 0 & -{\bar{z}}_{4} & 0 & {\bar{x}}_{4}\\ 0 & 0 & -1 & -{\bar{y}}_{5} & {\bar{x}}_{5} & 0\\ 0 & 0 & 1 & {\bar{y}}_{6} & -{\bar{x}}_{6} & 0 \end{array}\right]\left[\begin{array}{c} a_{px}\\ a_{py}\\ a_{pz}\\ \alpha_{x}\\ \alpha_{y}\\ \alpha_{z} \end{array}\right]$$

Assuming that the center of gravity of the accelerometer is located at the center of the upper surface of the panel, 6 mm from the installing surface, and the mass of the individual acceleration measurement component is *m*. The center of gravity positions of the six accelerometers on the platform float can be described as:

(33) $$\left\{ \begin{array}{l} {\vec{\rho }}_{1}={\left[\begin{array}{ccc} x_{0} & y_{1} & z_{1} \end{array}\right]}^{T},\;{\vec{\rho }}_{2}={\left[\begin{array}{ccc} -x_{0} & y_{2} & z_{2} \end{array}\right]}^{T},\;{\vec{\rho }}_{3}={\left[\begin{array}{ccc} x_{1} & y_{0} & z_{3} \end{array}\right]}^{T}\\ {\vec{\rho }}_{4}={\left[\begin{array}{ccc} x_{2} & -y_{0} & z_{4} \end{array}\right]}^{T},\;{\vec{\rho }}_{5}={\left[\begin{array}{ccc} x_{3} & y_{3} & z_{0} \end{array}\right]}^{T},\;{\vec{\rho }}_{6}={\left[\begin{array}{ccc} x_{4} & y_{4} & -z_{0} \end{array}\right]}^{T} \end{array}\right.$$

According to the definition of barycenter and product of inertia, we can obtain the Equation (34) as follows:

(34) $$\left\{ \begin{array}{l} \sum myx=m\left(y_{1}x_{0}-y_{2}x_{0}+x_{1}y_{0}-x_{2}y_{0}+x_{3}y_{3}+x_{4}y_{4}\right)=0\\ \sum mxz=m\left(x_{0}z_{1}-x_{0}z_{2}+x_{1}z_{3}+x_{2}z_{4}+x_{3}z_{0}-x_{4}z_{0}\right)=0\\ \sum myz=m\left(y_{1}z_{1}+y_{2}z_{2}+y_{0}z_{3}-y_{0}z_{4}+y_{3}z_{0}-y_{4}z_{0}\right)=0\\ \sum mx=m\left(x_{1}+x_{2}+x_{3}+x_{4}\right)=0\\ \sum my=m\left(y_{1}+y_{2}+y_{3}+y_{4}\right)=0\\ \sum mz=m\left(z_{1}+z_{2}+z_{3}+z_{4}\right)=0 \end{array}\right.$$

The length, width, and height of the mounting base of accelerometers are 52 mm, 52 mm and 75.84 mm. Therefore, *x*_0_ = 20 mm, *y*_0_ = 20 mm and *z*_0_ = 31.92 mm. To make ${\bar{y}}_{6}-{\bar{y}}_{5}$, ${\bar{x}}_{5}-{\bar{x}}_{6}$, ${\bar{z}}_{3}-{\bar{z}}_{4}$, ${\bar{x}}_{4}-{\bar{x}}_{3}$, ${\bar{z}}_{2}-{\bar{z}}_{1}$ and ${\bar{y}}_{1}-{\bar{y}}_{2}$ as large as possible, we take the value as follows:

(35) $$\left\{ \begin{array}{l} 20x_{1}+20y_{1}+x_{3}y_{3}=0\\ 31.92x_{3}+20z_{1}+x_{1}z_{3}=0\\ 31.92y_{3}+20z_{3}+y_{1}z_{1}=0 \end{array}\right.$$

For $\left(\begin{array}{cc} y_{1} & z_{1} \end{array}\right)$, $\left(\begin{array}{cc} x_{1} & z_{3} \end{array}\right)$ and $\left(\begin{array}{cc} x_{3} & y_{3} \end{array}\right)$ we take *x*_3_ = 8 mm, *y*_3_ = −8 mm and *y*_1_ = 12 mm according to the space of base installing. And we can solve the value that *x*_1_ = −8.8 mm, *z*_3_ = 16.162 mm and *z*_1_ = −5.6572 mm. The position of measuring point can be considered approximatively as the position of the barycenter of accelerometer, and we can find the rank of the coefficient matrix of Equation (32) to be 6 and the condition number to be 1.5599 × 10^−5^. So, the coefficient matrix of Equation (32) is invertible and well-conditioned. Thus, the acceleration calculation model is obtained.

## 5. Analysis of Electromagnetic Characteristics

**Figure 11:**
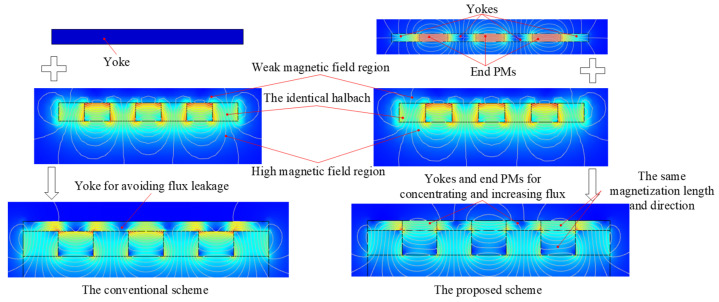
Comparison of design concepts of two schemes.

The main idea of improving the magnetic characteristics of the proposed scheme is to concentrate and increase the flux in the back region of halbach array, as shown in Figure 11. We know that the magnetic field at the back region of the traditional halbach array is small. In the conventional design process, the yoke is applied to reduce the magnetic resistance and leakage. The main innovation in the paper is to combine end PMs and yoke to concentrate and increase the flux in the back region to improve magnetic field distribution. This is the main difference between the design concepts of the two schemes. The magnetization length and direction of end PMs are the same as those of the horizontally magnetized PMs of halbach array. To facilitate assembly, we use an integral PM to replace the end PM and the horizontal PM. To highlight the improvement performance of the new structure, the magnetic flux density distribution, driving force and torque of the proposed scheme are investigated and compared with that of the conventional one by FEM.

### 5.1. Magnetic Flux Density

**Figure 12:**
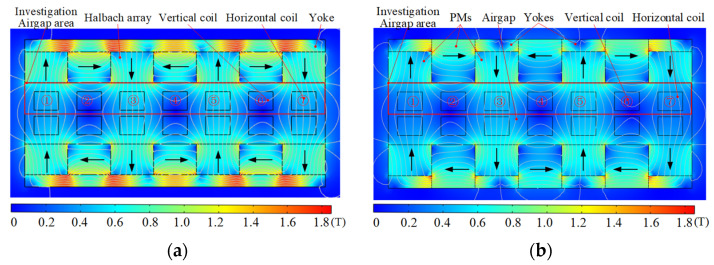
Flux nephogram of the 2-DOF electromagnetic actuators: (**a**) Conventional halbach array; (**b**) Improved halbach array.

**Figure 13:**
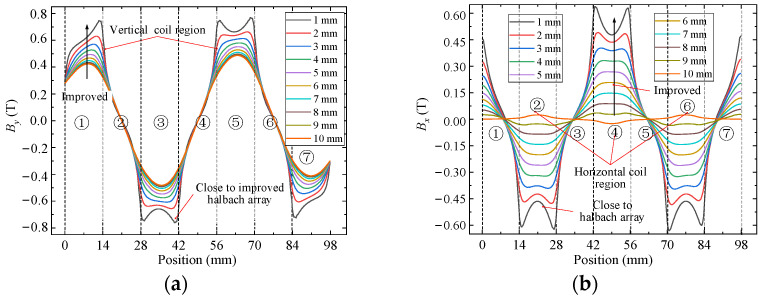
Airgap flux density distribution of conventional halbach array scheme: (**a**) *y* component; (**b**) *x* component.

**Figure 14:**
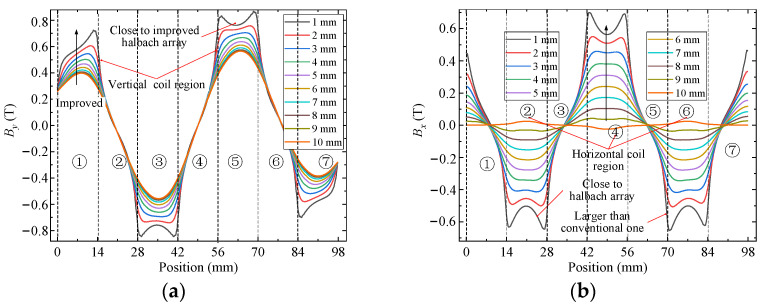
Airgap flux density distribution of improved halbach array scheme: (**a**) *y* component; (**b**) *x* component.

FEM is used to analyze and compare flux density characteristics of conventional and improved halbach array schemes, as shown in Figure 12. The arrangement and magnetization direction of PMs are the same in two schemes, respectively, which indicates that they have the same magnetic circuits. There is obvious magnetic saturation in the yoke part of the traditional scheme, while the new scheme effectively alleviates the magnetic saturation of the yoke because of the unique structure of PM and yoke arrays. From Equation (8), airgap magnetic field characteristics determine the driving force of the 2-DOF electromagnetic actuators. Because the magnetic fields in the upper and lower parts of the airgap are symmetrically distributed, only the upper part of the airgap is compared. *x* and *y* components of airgap flux density of two schemes are calculated by FEM, as shown in Figure 13 and Figure 14. The *x* and *y* components present a sinusoidal distribution in the center of the airgap, and the magnetic field near the PM region increases significantly. The airgap magnetic density distribution of the two schemes is non-uniform. In regions ①, ③, ⑤ and ⑦, the sum of *y* components is large and the sum of *x* components is small. In regions ②, ④ and ⑥, the sum of *x* components is large and the sum of *y* components is small. Compared with the traditional halbach array, the *y* components in region ③ and ⑤ and the *x* components in region ②, ④ and ⑥ of the new scheme are improved significantly. Therefore, the improved halbach array has the advantages of strengthening the airgap magnetic field and alleviating the magnetic saturation in the yoke.

### 5.2. Electromagnetic Force

**Figure 15:**
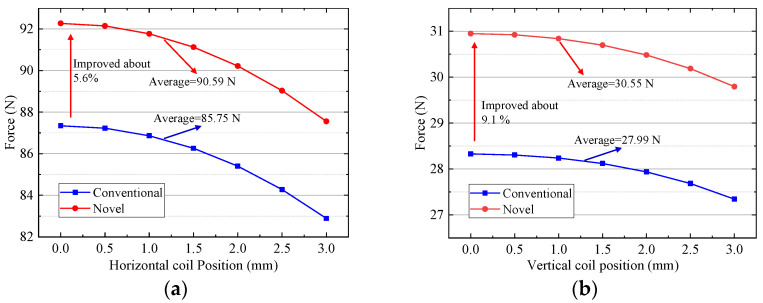
Driving force versus position in *x* direction: (**a**) Horizontal coil; (**b**) Vertical coil.

**Figure 16:**
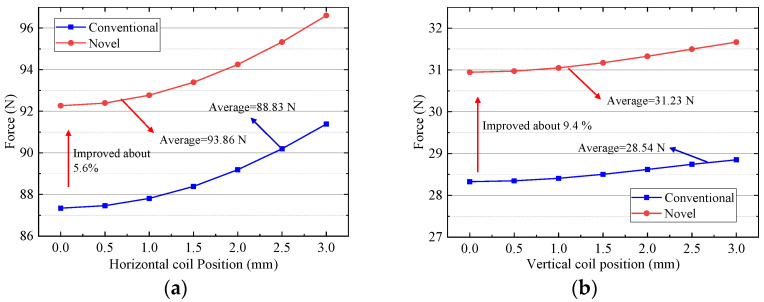
Driving force versus position in *y* direction: (**a**) Horizontal coil; (**b**) Vertical coil.

Under the control current of 1 A, driving force variation with position and deflection is analyzed by FEM. As shown in Figure 15 and Figure 16, the horizontal and vertical driving forces change obviously due to inhomogeneous magnetic field distribution. Under the same current, the horizontal driving force is larger than the vertical driving force, because the turns of horizontal coils are more than those of the vertical coils. Figure 15 shows that the driving force produced horizontal and vertical coil decreases with the increase of the coil movement in the *x* direction. The average horizontal force of the improved halbach array is 90.59 N, which is 5.6% larger than 85.75 N of the conventional one. That is because the *y* component of magnetic flux density in region ③ and ⑤ of improved scheme increase. The average vertical force of the improved halbach array is 30.55 N, which is 9.1% larger than 27.99 N of the conventional one. The reason is that the *x* component of magnetic flux density in regions ②, ④ and ⑥ of improved scheme increases dramatically. Because the improvement *x* component of flux density is more than that of *y* component, the enhancement of vertical force is greater than that of horizontal force. Figure 16 shows that the driving force produced horizontal and vertical coil improves with the increase of the coil movement in the *y* direction. The reason is that the magnetic field acting on the coil increases with the coil close to the PMs. The average vertical force of the improved halbach array is 93.86 N, which is 5.6% larger than 88.83 N of the conventional one. That is because the *y* component of magnetic flux density in region ③ and ⑤ of improved scheme increase. The average vertical force of the improved halbach array is 31.23 N, which is 9.4% larger than 28.54 N of the conventional one. The reason is that the *y* component of magnetic flux density in regions ②, ④ and ⑥ of improved scheme increases dramatically. So, compared with the conventional one, the horizontal and vertical driving forces of the new halbcah array scheme improve markedly when the windings move in the *x* and *y* directions.

The change of effective length of the winding in the inhomogeneous magnetic field results in the fluctuation of the driving force. The ripple ratio, *η*, is introduced to evaluate fluctuation of driving force with the movement of the coil, as follow:

(36) $$\eta =100\%\times \left|\left(F-F_{\mathrm{avg}}\right)/F_{\mathrm{avg}}\right|$$

**Figure 17:**
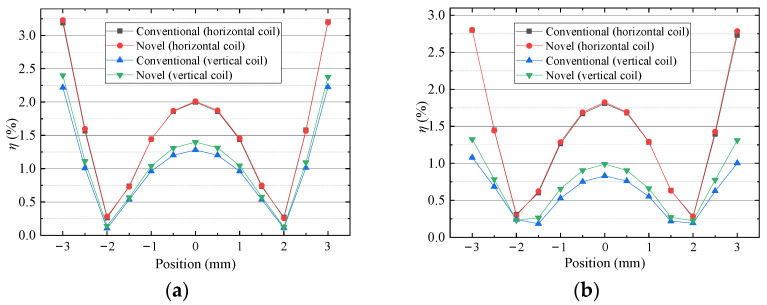
Ripple ratio versus position: (**a**) in *x* direction (**b**) in *y* direction.

Figure 17 shows that there is little difference in the ripple ratios between the two schemes and the ratios are less than 3.5%, which means that force fluctuation is small when the coils move in the *x* or *y* direction. Because the uniformity of *x* component of flux density is greater than that of the *y* component, the fluctuation of vertical force is better than that of horizontal force in the *x* or *y* direction. So, the driving force is stable in a small range of motion, ±3 mm, in the *x* or *y* direction.

**Figure 18:**
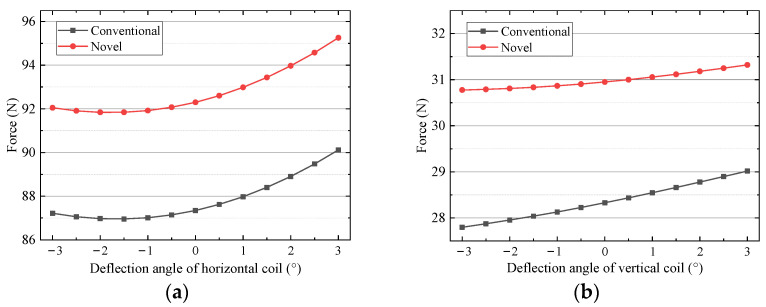
Driving force versus deflection around *z*-axial: (**a**) horizontal coil (**b**) vertical coil.

**Figure 19:**
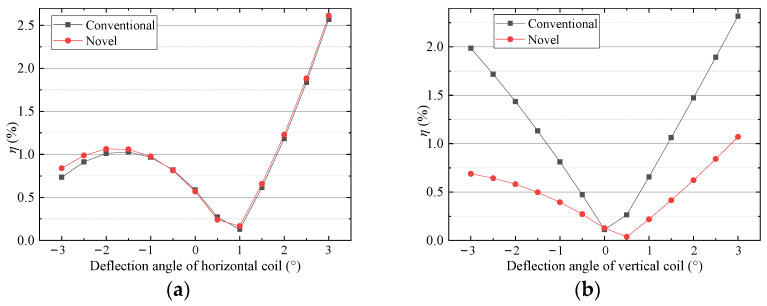
Ripple ratio versus deflection: (**a**) horizontal coil (**b**) vertical coil.

FEM is also used to analyze the driving force when the mover deflects around the *z*-axis. As shown in Figure 18, the horizontal driving force increase with the deflection of the mover. That is because the windings approach the surface of the pole of permanent magnets and the magnetic field acting on the coils increases. Figure 19 shows that the ripple ratios of the horizontal force of the two schemes are less than 2.6%. The ripple ratio of the vertical force of the new scheme is less than 1.1%, which is better than 2.7% of the conventional one. So, the driving force output is stable when the mover deflects.

**Figure 20:**
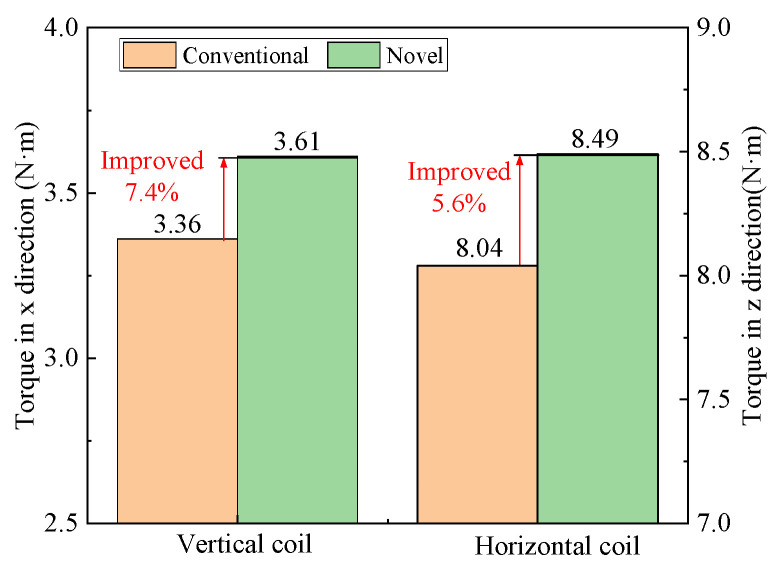
Comparison of torques of two halbach array schemes.

The torque output of two schemes is compared by FEM under the current of 1 A, as shown in Figure 20. Because the horizontal force is higher than the vertical force, the torque in the z direction is larger than that in the *x* direction under the same current. The torque in the *x* direction of the improved scheme is 3.61 N·m, which is 7.4% higher than 3.36 N·m of the conventional one. The torque in the *z* direction of the improved scheme is 8.49 N·m, which is 5.6% higher than 8.49 N·m of the conventional one. So, the performance of torque output of the new halbach array is improved effectively.

**Figure 21:**
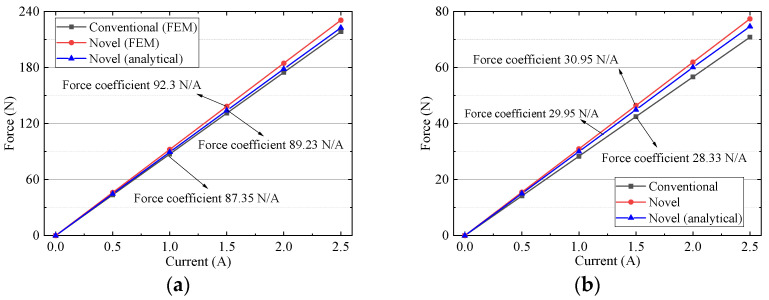
Driving force versus current: (**a**) horizontal coil (**b**) vertical coil.

Driving force variation with control current is studied by FEM, as shown in Figure 21. The driving force is proportional to the current. Analytical results of horizontal and vertical force coefficient are 89.23 N/A and 29.95 N/A, which are smaller than 92.3 N/A and 30.95 N/A of FEM results. The maximum error is less than 3.5%, which indicates that the analytical results agree well with FEM results and it is proved to be reliable. The horizontal force coefficient of the new scheme, 92.3 N/A, is 5.7% larger than 87.35 N/A of the conventional one. The vertical force coefficient of the new scheme, 30.95 N/A, is 9.2% larger than 28.33 N/A of the conventional one. That is because the magnetic field of the new scheme is greater than that of the conventional one.

**Figure 22:**
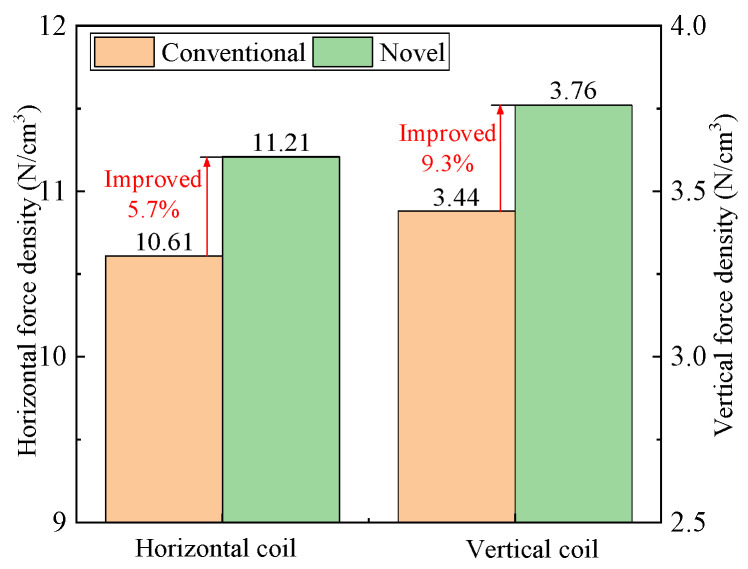
Comparison of driving force density of two models.

To highlight the merits of the new halbach array, the driving force densities of the two schemes are compared through the FEM. Driving force density can be defined as driving force/volume of halbach array. Figure 22 shows that the horizontal and vertical force densities of the new scheme are 11.21 N/cm^3^ and 3.76 N/cm^3^, which are larger than 10.61 N/cm^3^ and 3.44 N/cm^3^ of the conventional one.

Compared with the conventional halbach array scheme, the improved scheme achieves a higher force coefficient, torque, and force density, which is more suitable for the magnetic suspension platform. The functions and purposes of the 2-DOF electromagnetic actuator proposed in this paper are similar to those in reference [18]. The structure parameters and electromagnetic performance of two electromagnetic actuators are compared, as shown in Table 2. The motion range and deflection angle of the electromagnetic actuator in this paper are small than that of the reference value. The airgap flux density in the paper is much larger because of the improved halbach array structure. The horizontal and vertical force coefficients of 92.3 N/A and 30.95 N/A are greater than those of 4.027 N/A and 5.549 N/A due to more coil turns and a higher magnetic field. However, the temperature rise and power consumption are larger than those in reference. The volume of 687.5 cm^3^ is 53% lower than 1463 cm^3^ of the reference value.

## 6. Conclusions

A novel 2-DOF electromagnetic actuator with an improved halbach array is proposed for the magnetic suspension platform. The translation stroke is 3 × 3 × 3 mm, the overall dimension is 110 mm × 125 mm × 50 mm and the deflection angle is ±3°. The mathematical model of the magnetic field, electromagnetic force and torque are established by subdomain method. The magnetic field distribution, the driving force, and torque are analyzed through the FEM. Compared with the conventional halbach array scheme, the magnetic field is improved, and the magnetic saturation of the yoke is relieved. The horizontal and vertical force coefficients are 92.3 N/A and 30.95 N/A, which indicates high force output of the electromagnetic actuator is realized. Compared with the conventional similar 2-DOF electromagnetic actuator, the force coefficient and torque of the actuator in this paper are larger. The electromagnetic model, position measurement model of PSD, accelerometer measurement model, and electromagnetic analytical results in the paper provide support for the design and analysis of the 2-DOF electromagnetic actuator with high driving force coefficients.

## Figures and Tables

**Table 1:** The relationship between motion of the platform and driving forces of the actuators.

Movement of the Platform	Driving Force
Translation in *x*-axis direction	Horizontal driving forces from actuators 2 and 4
Translation in *y*-axis direction	Horizontal driving forces from actuators 1 and 3
Translation in *z*-axis direction	Vertical driving forces from actuators 1–4
Deflection around *x*-axis	Vertical driving forces from actuators 2 and 4
Deflection around *y*-axis	Vertical driving forces from actuators 1 and 3
Rotate around *z*-axis	Horizontal driving forces from actuators 1–4

**Table 2:** Comparison of the parameters of the actuators.

Parameters	In This Paper	In the Reference
Wire diameter	0.4 mm	1 mm
Vertical coils	2 × 300 turns	2 × 182 turns
Horizontal coils	3 × 300 turns	108 turns
Volume	687.5 cm^3^	1463 cm^3^
Maximum continuous current	2.5 A	2 A
Average Magnetic flux density	0.423 T	0.33 T
Vertical force coefficient	30.95 N/A	5.549 N/A
Horizontal force coefficient	92.3 N/A	4.027 N/A
Working range	3 × 3 × 3 mm	5 × 5 × 5 mm
Rotation	3° × 3° × 5°	10° × 10° × 10°

## Data Availability

Not applicable.
